# Short-Term Effect of Orthodontic Treatment with Clear Aligners on Pain and sEMG Activity of Masticatory Muscles

**DOI:** 10.3390/medicina57020178

**Published:** 2021-02-19

**Authors:** Alessandro Nota, Silvia Caruso, Shideh Ehsani, Gianmaria Fabrizio Ferrazzano, Roberto Gatto, Simona Tecco

**Affiliations:** 1 Dental School, Vita-Salute San Raffaele University and IRCCS San Raffaele Hospital, 20132 Milan, Italy; shideh_ehsani@yahoo.it (S.E.); tecco.simona@hsr.it (S.T.); 2 Department of Life, Health and Environmental Science, University of L’Aquila, 67100 L’Aquila, Italy; silvia.caruso@univaq.it (S.C.); roberto.gatto@cc.univaq.it (R.G.); 3 Department of Neuroscience, Reproductive and Oral Science, School of Paediatric Dentistry, University of Naples “Federico II”, 34012 Naples, Italy; gianmariafabrizio@yahoo.it; 4 Unesco Chair in Health Education and Sustainable Development, University of Naples “Federico II”, 34012 Naples, Italy

**Keywords:** interceptive orthodontics, removable orthodontic appliances, electromyography, masticatory muscles, orthodontics

## Abstract

*Background and objectives:* The aim of this study is to evaluate mandibular elevator muscles activity and pain on palpation in the early stages of orthodontic treatment with clear aligners using surface electromyography (sEMG). *Materials and methods:* Surface electromyography (sEMG) activity and pain level on muscle palpation of masseter and anterior temporalis muscles were recorded in a sample of 16 adult subjects (aged 18–32 years; mean 22.5 +/− 3.5 SD) undergoing orthodontic treatment with clear aligners before the treatment (T0), after 1 month of treatment (two clear aligners) (T1), and after 3 months of treatment (T2) (six clear aligners). A chi-square test for nominal data, a Friedman test, and a Wilcoxon-signed rank test as post hoc analysis were applied. *Results:* No statistically significant differences in muscular pain were observed. At T1, the sEMG activity of masseter muscles at mandibular rest position showed a statistically significant reduction, but after 3 months (T2), the data appeared similar to T0 (*p* = 0.03 and *p* = 0.02). *Conclusions:* During the treatment with clear aligners, subjects could experience an initial reduction in the masseter basal activity after 1 month of treatment. This effect tends to decrease to baseline levels after 3 months of therapy.

## 1. Introduction

In the last decades, an increasing number of adult patients have been looking for orthodontic treatment and expressed a desire for aesthetic and comfortable alternatives to conventional fixed appliances [1,2]. Clear aligners are an orthodontic system that has been introduced as an answer to this request. Recent systematic reviews have shown that orthodontic treatment with clear aligners has been effective in obtaining a proper alignment and leveling of the dental arches during the last decade, even in more complex cases and in children or adolescents [3,4]. Moreover, compared to treatment with fixed appliances, orthodontic treatment with removable appliances and especially clear aligners has shown better preservation of periodontal indexes and oral hygiene, related to a lower risk of periodontal damage or enamel demineralization and caries [5,6,7,8].

The scientific literature shows that orthodontic treatment and occlusal appliances can affect masticatory muscle activation [9,10,11,12]. For example, in patients with myofascial pain syndrome, teeth alignment with multi-bracket devices seems to improve, although not resolve, signs and symptoms of masticatory muscle pain [13] and the use of removable retainer appliances with dental coverage is able to reduce the basal activity of the anterior temporalis muscle [10]. Furthermore, in subjects with temporomandibular joint internal derangement, an occlusal appliance affects the masseter and anterior temporalis activation pattern [11] and after 1 month of treatment with occlusal splints, subjects with myofascial pain syndrome showed a reduced degree of fatigue of the masticatory muscles during maximal voluntary clenching [14]. As temporomandibular disorders (TMD) have a complex multifactorial etiology [15,16], the reaction of muscles is dependent not only on the dental relationship but also on many other factors.

Some previous studies have analyzed the impact of functional and orthodontic appliances on masticatory muscle activity [12,17,18,19], and a recent study [20] also analyzed the impact of wearing clear aligners on masticatory muscle activity, but to the best of the authors’ knowledge, there are no studies in literature on the effects of orthodontic treatment with these appliances on masticatory muscle activity and muscular pain on palpation, and there is even a common belief that clear aligners are not indicated in subjects with signs or symptoms of TMD [21]. The aim of this preliminary study was to evaluate the short-term changes in mandibular elevator muscle activity and pain on palpation during the early stages of orthodontic treatment with clear aligners. The hypothesis is that patients undergoing orthodontic treatment could experience a variation in muscular activity and an associated increase in masticatory muscle pain.

## 2. Materials and Methods

### 2.1. Subjects

A total of 16 subjects (8 females and 8 males; aged 18–32 years; mean age 22.5 +/− 3.5 years) were included in the sample of this preliminary study according to the following inclusion criteria: presence of class-I malocclusion and crowding. The exclusion criterion was the presence of TMD diagnosis according to Research Diagnostic Criteria for Temporomandibular Disorders (RDC/TMD).

### 2.2. Study Procedure and Data Analysis

Informed consent was obtained from each patient. The study was conducted according to the guidelines of the Declaration of Helsinki and approved by the Ethics Committee of the University of L’Aquila (Document DR206/2013). Subjects underwent orthodontic treatment with clear aligners (Invisalign^®^, Align Technology, San José, CA, USA), the aligners being changed every 2 weeks and with a monthly treatment follow-up; surface electromyographic (sEMG) activity and pain on muscular palpation of masseter and anterior temporalis muscles were recorded before the beginning of orthodontic treatment (T0), after 1 month of treatment (two clear aligners) (T1), and after 3 months of treatment (six clear aligners) (T2).

### 2.3. Muscular Pain

At T0, T1, and T2, palpation of the masticatory muscles area (masseter and anterior temporalis) was performed. Subjects were invited to assess their pain on a scale of 0 to 3, where 0 indicated the absence of pain, 1 indicated a complaint feeling, 2 meant light pain, and 3 denoted full-blown pain. A single expert operator made all the visits and carried out the palpation at T0, T1, and T2. Data were collected as the number of subjects with pain on palpation for each level of pain. T0, T1, and T2 data were then compared by using the chi-square test.

### 2.4. sEMG Recording

The sEMG activity of masticatory muscles was recorded bilaterally, (i) with the mandible at rest position and (ii) during maximal voluntary clenching (MVC). The masticatory muscles’ sEMG potentials of masseter and anterior temporalis were measured by a 4-channel Key-Win EMG (Biotronic, San Benedetto del Tronto, Italy), with a passband of 25–1500 Hz. Duo-Trode bipolar surface rectangular electrodes (10 × 5 mm^2^), with a fixed interelectrode distance of 20 mm, were used. For the assessment of maximal voluntary clenching, a protocol previously published was applied [22]. All subjects were instructed to close the mandible in habitual occlusion as forcefully as possible. Movement patterns were conducted for at least three repetitions to ascertain stability. The initial movement patterns were eliminated as the “learning” sequence, as they were frequently observed to be dissimilar to the other two repetitions. The third movement was considered the most stable. Therefore, the root mean square (RMS) data were obtained. To standardize the sEMG recordings, all subjects were asked to maintain a comfortable posture, relax their arms by their sides, look straight ahead, and make no head or body movements during the test. The tested areas were masseter and anterior temporalis muscles on both sides. The sEMG recording time for each analysis was 15 s, and values were expressed in microvolts per second (μV s^−1^) [22]. The sample size was a priori calculated on the basis of a pilot study for a significance level of 0.05 to achieve a sample power of minimum 80% on the sEMG recordings considered as primary outcome; a minimum of 14 subjects was required. Due to the small number of subjects, and considering the not-normal distribution according to the Shapiro–Wilk test, data on sEMG activity were collected as the median and the twenty-fifth (Q1) and seventy-fifth percentiles (Q3) and the Friedman test and the Wilcoxon-signed rank test as post hoc analysis were used to test the differences among T0, T1, and T2 recordings. The significance level was set at *p* = 0.05. All the statistical analyses were conducted using SPSS software (version 24; IBM, Armonk, NY, USA).

## 3. Results

A total of 16 subjects were analyzed for muscular sEMG activity and pain. Data were recorded, and sEMG data were analyzed with descriptive statistics expressed as median and quartiles.

### 3.1. Muscular Pain

The percentage of subjects with muscular pain on palpation at T0 was 12.5% (2 subjects of 16 subjects with Level 2). At T1, the percentage of subjects with muscular pain on palpation became 25% (4 subjects of 16 subjects with Level 2). At T2, only 1 subject reported muscular pain (Level 2). No statistically significant differences were observed between T0, T1, and T2 values. (Table 1).

### 3.2. sEMG Activity

No difference was observed in the sEMG activity of the anterior temporalis muscle over time. At T1, the subjects showed a significant reduction in the sEMG activity of the masseter muscle at mandibular rest position compared to T0 (Table 2). No differences were observed during maximal voluntary clenching (Table 3).

## 4. Discussion

This study evaluated sEMG activity and pain on muscular palpation of the masseter and anterior temporalis muscles before the beginning of orthodontic treatment (T0), after 1 month of treatment (two aligners) (T1), and after 3 months of treatment (six aligners) (T2) in 16 adult subjects (aged 18–32 years), characterized by class-I malocclusion and crowding and with no TMD history. To the best of the authors’ knowledge, this is the first study that analyzes masticatory muscle activity and pain on palpation during 3 months of orthodontic treatment with clear aligners.

For the present investigation, all subjects were treated using only one aligner system in order to eliminate potentially associated confounding factors, including differences in plastic aligner material composition, flexibility, thickness, and force activation and consequent stress-relaxation differences, because the forces exerted by orthodontic clear aligner appliances can be influenced by the mechanical properties of their material.

Muscular pain should be prevented, especially in patients with TMD, and it can reduce patient compliance, compromising the effectiveness, comfort, and satisfaction of orthodontic treatment and determining negative effects on temporomandibular joint health. A recently published study [23] showed that the adaptation to clear aligner therapy in terms of muscle soreness is acceptable and follows the same pattern in terms of trending toward baseline levels after 4 weeks. In the present study, instead, pain on muscular palpation of an expert operator remained stable without significant variations during 3 months of orthodontic treatment.

Looking at the findings of the present study, it seems that active tooth movements with aligners do not cause substantial muscular pain in the short term.

At the beginning of the treatment (T1), patients wearing clear aligners experienced a statistically significant reduction in the sEMG activity of their masseter muscles at mandibular rest position. All the differences in the basal activity of masseter muscles recorded at T1 disappeared at T2 after 3 months of orthodontic treatment with clear aligners. Furthermore, according to the classical theory by Møller [24], this short-time effect of the aligners could be explained by a slight change in the position of the mandible generating an altered modulation of the masseter muscle [25]. Occlusal stability seems to be associated with neuromuscular function [26,27], and the presence of the appliance could have caused an early and temporary effect on the proprioceptive information given to the central nervous system (CNS) [28]. Interestingly, a previous similar study [20] performed in a limited time of 4 weeks observed an increase in the masticatory muscle activity in the first 2 weeks and a return to the baseline after 4 weeks. Unfortunately, this previous study was limited by the reduced observation time, which prevented tooth movements of more than 0.5 mm as only two active aligners were applied, and by the use of portable sEMG probes that were positioned by the patients themselves. This could have caused a significant bias in the obtained results as a patient has no experience in achieving the correct position of sEMG electrodes.

The decrease in masticatory muscle sEMG values expresses a decrease in muscular activity that could be related to the introduction of occlusal changes. Aligners could slightly alter occlusal relationships and vertical dimension, leading to temporary lower muscular activity that decreases after the first month to the initial values. The short-term change in muscle activity is evident between T0 and T1 stages (approximately after 1 month of treatment) but goes back to baseline values at T2 (approximately after 3 months of treatment), appearing as only a temporaneous increase in muscular activity in the first weeks of aligners.

On the other hand, aligners seem not to produce any effect of muscle pain. Thus, maybe they could be used in patients who have TMD if this was previously managed with proper therapy, but it should be also considered that, as explained before, masticatory muscle activation is related not only to dental occlusion but also to many other factors [15,16,17,18].

As clear aligners represent an alternative to the multi-brackets fixed device, it would be interesting to compare the results obtained with data obtained from orthodontic multi-brackets appliances. In literature, however, no studies of this kind are present. A previous study made by Tecco et al. evaluated the influence of fixed multi-bracket device treatment on muscle pain in a sample of adult patients with myofascial pain syndrome (MPS), showing an improvement in, although no resolution of, the signs and symptoms of MPS (a significant temporomandibular joint and masticatory muscle pain reduction) at the end of the dental alignment and dental class correction [13]. In the present study, the observation time was limited to 3 months of treatment and the subjects were not affected by TMD.

Considering that the present study showed no alteration in the muscular activity and pain of masticatory muscles during a period of 3 months of orthodontic treatment, this study seems to suggest that future studies should be performed to analyze if clear aligner orthodontic treatment could be not contraindicated in subjects affected by TMD, analyzing the effects of clear aligners on the muscular activity of these subjects [21]. Due to the limited follow-up of this study, an effect of orthodontic treatment with clear aligners after 3 months of treatment cannot be excluded. Further studies are needed to deeply understand the phenomena, and effects of the recently introduced weekly aligner change should also be examined. Further evaluations could be of interest to possibly identify—before the beginning of treatment—individuals potentially more sensitive to pain and discomfort during orthodontic therapy.

## 5. Conclusions

During orthodontic treatment with clear aligners, the subjects experienced an initial reduction in masseter basal activity after 1 month of treatment. This effect tends to decrease to baseline levels after 3 months of therapy.

## Figures and Tables

**Table 1:** Number of subjects for each level of pain at T0, T1, and T2.

	Level 0 (*n*)	Level 1 (*n*)	Level 2 (*n*)	Level 3 (*n*)	
**T0**	6	8	2	0	T1 *p* = 0.26 NS T2 *p* = 0.71 NS
**T1**	4	8	4	0	
**T2**	7	8	1	0	

NS: result not significant at *p* > 0.05. T0: before the beginning of orthodontic treatment. T1: after 1 month of treatment (two clear aligners). T2: after 3 months of treatment (six clear aligners). *n*: number of subjects.

**Table 2:** Statistical analysis of the surface electromyography (sEMG) recording of masseter and anterior temporalis muscles (μV s^−1^) at mandibular rest position (*n* = 16).

	T0		T1			T2			
	Median	Q1/Q3	Median	Q1/Q3	T0 vs. T1 (Wilcoxon)	Median	Q1/Q3	T0 vs. T2 (Wilcoxon)	Friedman Test
**Anterior temporalis (L)**	9.1	4.15/9.7	9.2	4.5/10.2	NA	9.2	5.5/11.5	NA	NS
**Anterior temporalis (R)**	9.3	5.2/9.9	8.1	5.2/8.6	NA	9.2	5.5/10.5	NA	NS
**Masseter (L)**	6.1	3.9/7.7	4.7	2.8/6.2	*	5.1	3.3/7.3	NS	*p* = 0.03
**Masseter (R)**	6.2	3.8/7.5	4.7	2.7/7.4	*	5.4	3.2/7.5	NS	*p* = 0.02

* *p* < 0.05; significant difference between T0 and T1 in the test group. NS: no significant difference. NA: not applied. Q1: the twenty-fifth percentiles. Q3: the seventy-fifth percentiles.

**Table 3:** Statistical analysis of the sEMG recording of masseter and anterior temporalis muscles (μV s^−1^) during maximal voluntary clenching in the test group (*n* = 16).

	T0		T1		T2		
	Median	Q1/Q3	Median	Q1/Q3	Median	Q1/Q3	Friedman Test
**Anterior temporalis (L)**	71.2	45.2/98.5	75.2	43.2/94.3	68.2	46.5/91.3	NS
**Anterior temporalis (R)**	73.0	46.3/94.8	75.2	44.5/99.2	71.3	49.5/90.5	NS
**Masseter (L)**	54.6	31.3/75.1	53.9	36.5/68.2	54.4	33.4/71.8	NS
**Masseter (R)**	56.2	35.1/76.3	55.9	37.1/70.4	54.6	34.2/64.6	NS

## Data Availability

The data that support the findings of this study are available from the University of L’Aquila, but restrictions apply to the availability of these data, which were used under license for the current study, and so are not publicly available. Data are however available from the authors upon reasonable request and with permission of the University of L’Aquila partner.
